# Evaluation of Cytotoxicity of the Dental Materials TheraCal LC, TheraCal PT, ApaCal ART and Biodentine Used in Vital Pulp Therapy: In Vitro Study

**DOI:** 10.3390/dj12080249

**Published:** 2024-08-07

**Authors:** Barbora Novotná, Pavel Holík, Yuliya Morozova, Matej Rosa, Adéla Galandáková, Kateřina Langová

**Affiliations:** 1 Institute of Dentistry and Oral Sciences, Faculty of Medicine and Dentistry, Palacký University and Faculty Hospital Olomouc, Palackého 12, 779 00 Olomouc, Czech Republic; 2 Department of Medical Chemistry and Biochemistry, Faculty of Medicine and Dentistry, Palacký University, Hněvotínská 3, 775 15 Olomouc, Czech Republic; 3 Department of Medical Biophysics, Faculty of Medicine and Dentistry, Palacký University, Hněvotínská 3, 775 15 Olomouc, Czech Republic

**Keywords:** cytotoxicity, calcium silicate, calcium phosphate, resin-modified, dental pulp, vital pulp treatment, deep caries, MTT assay

## Abstract

(1) Background: The aim of this study was to compare the cytotoxicity of selected resin-modified materials used in direct contact with the dental pulp (TheraCal LC, TheraCal PT, and ApaCal ART) with calcium silicate cement (Biodentine). (2) Methods: The mouse fibroblast Balb/3T3 cell line and the extracts of tested materials in four concentrations were used for the testing. An MTT assay was performed in three independent experiments with six replicates for each concentration of tested material. The cell viability (%) and cytotoxicity were expressed (cytotoxic effect is considered in cases where the cell viability is lower than 70%). The mean of the cell viability and the standard deviation were expressed for each material at all concentrations. ANOVA and Dunnet’s post hoc tests were used for the statistical analysis. All of these tests were performed at the 0.05 significance level. (3) Results: At all concentrations, the cell viability was statistically significantly lower (*p* ≤ 0.002) for all tested materials compared to Biodentine. ApaCal ART showed a high level of cytotoxicity at all concentrations (cell viability lower than 47.71%, *p* < 0.0001). The same result was found for TheraCal LC at concentrations of 100%, 50% and 25% and TheraCal PT at concentrations of 100% and 50%. TheraCal LC at a 10% concentration (cell viability 68.18%) and TheraCal PT at a 25% concentration (cell viability 60.63%) indicated potential cytotoxicity. TheraCal PT at a 10% concentration was not found to be cytotoxic (cell viability 79.18%, *p* = 0.095). (4) Conclusion: The resin-modified calcium silicate and calcium phosphate materials showed higher cytotoxic potential, so they should be used with caution when in direct contact with the dental pulp.

## 1. Introduction

In the case of deep or extremely deep caries lesions close to the dental pulp, there is a risk of the pulp chamber opening when excavating the carious tissues. There are several different procedures to manage these situations, which differ mainly in the depth of preparation and the chosen material. For better orientation in the indication of these procedures, in 2016, the International Caries Consensus Collaboration (ICCC) posted a statement in which they unified the terminology for carious lesions and their removal methods [1]. Based on this classification, in 2019, the European Endodontic Society published a recommendation describing all procedures aimed at maintaining the healthy dental pulp in the case of the treatment of deep and extremely deep caries lesions. These procedures are commonly labelled as a vital pulp therapy (vital pulp treatment, VPT) [2].

Vital pulp therapy is a summary of different restorative dental procedures aimed to maintain the vital dental pulp. Each of them is a combination of special types of excavation (non-selective carious tissue removal, selective carious removal to soft or firm dentine) and the application of different materials. VPT can be divided into indirect pulp capping, selective carious tissue removal in one stage, stepwise excavation, direct pulp capping, and partial and cervical pulpotomy [2].

It is recommended that a biocompatible material be used in all of these procedures. Although the main function of this material is a physical barrier to seal and prevent a connection between the dental pulp and oral cavity [3], it should ideally meet all or at least most of the required properties, such as the stimulation of reparative dentin formation, the maintenance of pulp vitality (biocompatibility and non-cytotoxicity), bactericidal or bacteriostatic properties, adhesion to dentin or other filling materials (hermetic sealing), mechanical and chemical resistance, remineralisation potential (fluoride or calcium ion release), and radiopacity [4,5].

The selection of the correct material for proper sealing and healing of the pulpal tissue is essential [6]. It is possible to use both composite material [7] and glass ionomer cement or calcium silicate cement [2] to cover the thin layer of dentin without dental pulp exposure. However, since the material is in direct contact with the vital dental pulp in half of the mentioned procedures, the most important parameters are a positive effect on hard-tissue barrier formation [8] and the facilitation of wounded pulp healing [9]. According to this information, the material should have as little cytotoxic effect on pulp cells as possible. For this reason, it is not appropriate to use a composite material here [2,10]. On the other hand, materials from the group of calcium silicate cements, which meet most of the required properties, mainly with a minimal level of cytotoxicity, are nowadays considered the gold standard in these indications [2,11].

Calcium silicate cements (hygroscopic dental cements, bioceramics [12]) have been available in the dental market since the 1990s [13]; thus, their properties and clinical applications are already relatively well researched [14]. With technological development, new materials containing calcium silicate or calcium phosphate components in different quantities appear in the dental market over time, as the manufacturers constantly want to improve them. They are available in different forms: as a powder and liquid for manual mixing, in the form of capsules for machine mixing, or even premixed versions. However, all of these forms have very similar properties [15,16]. Considering the limitations such as difficult manipulation and the relatively long setting time of classical calcium silicate cements [17], the aim of innovators is to eliminate these problems. Hence, there is a group of modified materials that are light-cured, which eliminates these difficulties during the treatment. Light-cured material must have some content of monomers in their composition. The manufacturer usually only states the presence of these ingredients in the product, but the quantity is often not declared. Therefore, it can only be assumed that the biocompatibility of these materials may decrease [10]. However, these materials are also declared as biocompatible materials, suitable for direct contact with dental pulp [18,19]. Due to the large number of materials with different compositions, it can be difficult for dentists to choose the most appropriate one for a given indication.

The aim of this study is to evaluate the cytotoxic effect of three groups of materials that differ in composition (light-cured calcium silicate cements, dual-cured calcium silicate cements, and light-cured calcium phosphate cements) and compare their cytotoxicity with the reference material, thus creating a comprehensive recommendation for general dentists.

Biodentine^TM^ (Septodont, Saint Maur des Fosses, France) is a classical calcium silicate material that was developed at the beginning of this millennium and can be included in the second-generation group as it is supplied in capsules and is machine-mixed in a precise powder to liquid ratio [20,21]. It has similar physical, chemical, and biological properties to other calcium silicate cements [22]. The main advantage is the precisely given mixing ratio, as well as the setting time of 12–15 min, that is gained by the admixture of calcium chloride [20,23]. Hence, the primary indication of this material is a dentine substitution [24] and is recommended for the procedures that preserve the vitality of the dental pulp [23]. For this reason, it was selected as a reference material for this in vitro study.

TheraCal LC^®^ (Bisco, Schaumburg, IL, USA) was chosen for this study as a representative of light-cured materials with calcium silicate particles. This material is premixed in the syringe with a cannula, and due to the admixture of the resin component, the setting is ensured by light-induced polymerisation. According to this, the handling and application of the material to the required area of the cavity is better than in the case of capsule-mixed Biodentine; moreover, the time of the whole procedure is shortened, as there is no necessity to wait for the material to set. It has been present in the dental market for over 10 years; thus, there are already some data available. It has shown good results mainly as a base material (compared with Dycal^®^ (Dentsply DeTrey GmbH, Konstanz, Germany)) in cases of indirect pulp capping [25,26].

TheraCal PT^®^ (Bisco, Schaumburg, IL, USA), the “successor” to TheraCal LC, was chosen as another material to be evaluated, as it is a dual-cured material. Therefore, it can be assumed that the principle of setting is different from purely light-cured materials in favour of the hydration of calcium silicate particles and positively affects the mineralisation of dentine [27]. Accordingly, it should improve the properties from the previous light-cured version. The material was introduced into the dental market less than five years ago. It is supplied in a dual syringe with an automix tip. As it is a relatively new material, its biological properties have been described in only a few studies. It has been proven that due to the different principle of setting, its release of Bisphenol A-glycidyl methacrylate and polyethylene glycol dimethacrylate was lower than in TheraCal LC [28]. According to this, the cytotoxicity effect should be lower in the case of the dual-cured material compared to the light-cured version.

ApaCal ART^®^ (Prevest DenPro, Bari Brahmana, India) was chosen as the third evaluated material, due to the content of calcium phosphate particles as the main component. It is also a resin-modified material, and it has the same form (a syringe with cannula) and principle of setting (light-cured) as TheraCal LC. As the material is relatively new, only a few studies have been described in recent years. These studies were focused mostly on the material’s microscopic and elemental characterisation [29], bond strength to dental adhesive systems [30,31], and radiopacity [32].

Null hypothesis H_0_: Resin-modified calcium silicate or calcium phosphate cements show the same level of cytotoxicity as classic calcium silicate cements.

Alternative hypothesis H_a_: Calcium silicate or calcium phosphate materials that are resin-modified show higher levels of cytotoxicity than classic calcium silicate cements.

## 2. Materials and Methods

### 2.1. Materials

The cytotoxicity of resin-modified materials with calcium silicate component, namely, TheraCal PT^®^ (Bisco, Schaumburg, IL, USA) and TheraCal LC^®^ (Bisco Schaumburg, IL, USA) and that with a calcium phosphate component, ApaCal ART^®^ (Prevest DenPro, Bari Brahmana, India), was evaluated in this in vitro study. Biodentine^TM^ (Septodont, Saint Maur des Fosses, France), a commonly used calcium silicate cement in this indication, was used as a reference material. The composition of these materials is specified in Table 1.

### 2.2. Preparation and Extraction of Test Materials

Discs with a diameter of 8 mm and a height of 1 mm were prepared from the tested materials in standard laboratory conditions. TheraCal PT, TheraCal LC, and ApaCal ART, materials presented in a premixed format, were injected into the sterile cylindrical moulds, light-cured (1000 mW/cm^2^, 2 mm distance, for the time according to the manufacturer’s instructions) using a Valo^TM^ lamp (Ultradent Products, Inc., South Jordan, UT, USA) and left to mature (37 °C, 24 h). Biodentine was mixed according to the manufacturer’s instructions, applied into the sterile cylindrical moulds, and left to ensure chemical setting (37 °C, 24 h). All materials were sterilised by UV radiation (15 min, both sides) before the extraction.

Extracts of all investigated materials were prepared according to the International Standard ISO 10993-5:2010 [36,37]. The test materials were put into a sterile tube (50 mL) and the appropriate amount (6 cm^2^/mL) of extraction medium (Dulbecco’s modified Eagle’s medium supplemented with foetal calf serum (5%, *v*/*v*), newborn calf serum (5%, *v*/*v*), streptomycin (100 U/mL), and penicillin (0.1 mg/mL)) was added. The tube was then incubated in a thermoblock (37 °C, 24 h) and the obtained extract (100%) was subsequently diluted with extraction medium to concentrations of 50%, 25%, and 10%. All 4 concentrations were used for cytotoxicity evaluation.

### 2.3. Evaluation of Cytotoxicity of Test Materials Using the MTT Assay

The cytotoxicity was evaluated using the mouse fibroblast Balb/3T3 cell line (clone A31, obtained from American Type Culture Collection, Manassas, VA, USA). The guidance for the test is in accordance with the International Standard ISO 10993-5:2010. The cells were grown in a 75 cm^2^ tissue culture flask in culture medium (Dulbecco’s modified Eagle’s medium supplemented with foetal calf serum (5%, *v*/*v*), newborn calf serum (5%, *v*/*v*), streptomycin (100 U/mL), and penicillin (0.1 mg/mL)) in a humidified atmosphere with 5% (*v*/*v*) CO_2_ at 37 °C.

**Figure 1:**
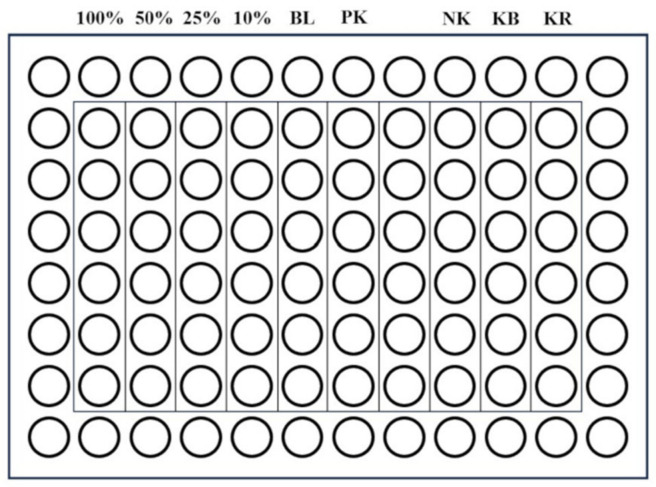
A 96-well plate (100%, 50%, 25%, and 10%—concentration of the material extract; BL—blank; PK—positive control; NK—negative control; KB—control of cells; KR—control of reagents).

For the experiment of each material, the cells were seeded on 96-well plates (200 µL/well) at a density of 0.8 × 10^5^ cells/mL and incubated in a humidified atmosphere (5% CO_2_, 37 °C) for 24 h. Then, the culture medium was removed and the extracts of the tested material (concentrations of 100%, 50%, 25%, and 10%) were applied on the cells together with appropriate controls—a positive control (cytotoxic substance—Triton X-100, 1% (*v*/*v*)), a negative control (biocompatible, non-cytotoxic material—extracts of polyvinyl chloride tube), a control of reagents (culture medium incubated at 37 °C, 24 h), and control cells (fresh culture medium) (Figure 1).

**Figure 2:**
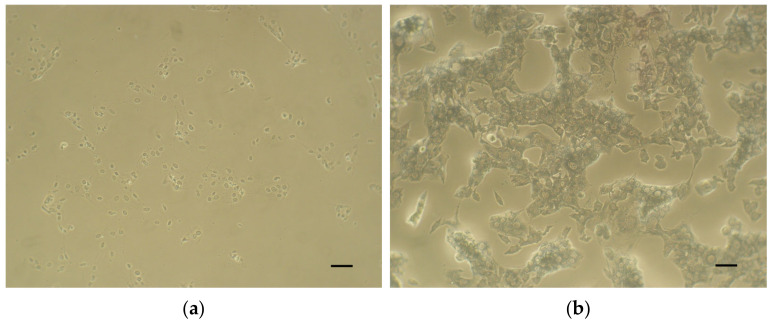
Microscopic images of control cell cultures (magnification: 100×, scale bar: 100 μm). (**a**) Positive control (cytotoxic substance—Triton X-100, 1% (*v*/*v*)). (**b**) Negative control (biocompatible, non-cytotoxic material—extracts of polyvinyl chloride tube).

**Figure 3:**
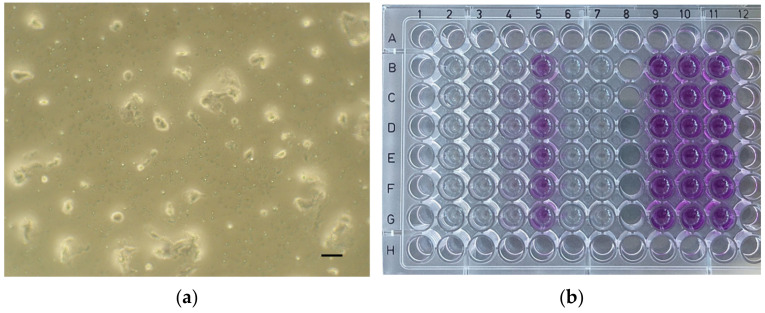
ApaCal ART. (**a**) Microscopic image of cell culture at 100% concentration (magnification: 100×, scale bar: 100 μm); (**b**) 96-well plate with ApaCal ART before the absorbance measurement.

**Figure 4:**
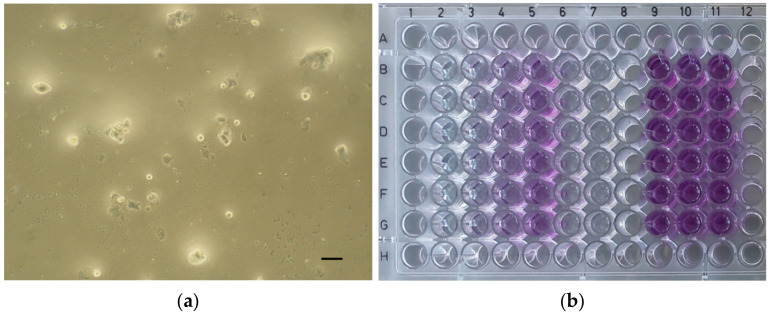
TheraCal LC. (**a**) Microscopic image of cell culture at 100% concentration (magnification: 100×, scale bar: 100 μm); (**b**) 96-well plate with TheraCal LC before the absorbance measurement.

**Figure 5:**
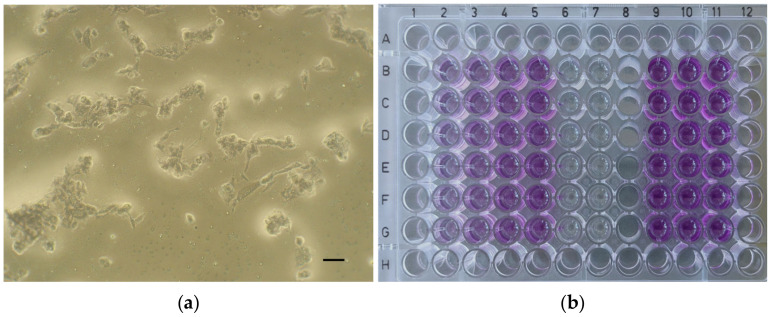
TheraCal PT. (**a**) Microscopic image of cell culture at 100% concentration (magnification: 100×, scale bar: 100 μm); (**b**) 96-well plate with TheraCal PT before the absorbance measurement.

**Figure 6:**
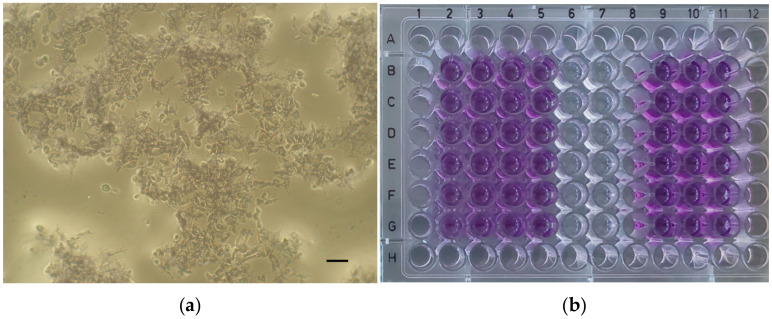
Biodentine. (**a**) Microscopic image of cell culture at 100% concentration (magnification: 100×, scale bar: 100 μm); (**b**) 96-well plate with Biodentine before the absorbance measurement.

Then, the plates were incubated in a humidified atmosphere (5% CO_2_, 37 °C) for 24 h. After the incubation period, the extracts of the tested material and the controls were removed and fresh culture medium supplemented with MTT (5 mg/mL; 100 µL) was applied to the cells (37 °C, 2 h). Then, the medium was removed, and the formazan crystals were dissolved in dimethyl sulfoxide (DMSO) with NH_3_ (1%, *v*/*v*). Microscopic images of the positive control (Figure 2a), negative control (Figure 2b), and the cell cultures of each material at a concentration of 100% (Figure 3a, Figure 4a, Figure 5a and Figure 6a) were captured with an Olympus CK2-TR microscope (Olympus optical CO., LTD, Tokyo, Japan) at a magnification of 100×. Subsequently, the absorbance of blue-coloured product was measured using a microplate reader (Sunrise Remote, Tecan, Grödig, Austria) at 570 nm (Figure 3b, Figure 4b, Figure 5b and Figure 6b).

Calculation of cell viability:

$$cell\;viability(\%)=\frac{mean\;A_{s}-mean\;A_{b}}{mean\;A_{c}-mean\;A_{b}}\times 100$$

A_s_—absorbance of sample (cells treated with extract of tested material);

A_b_—absorbance of blank (empty wells with DMSO);

A_c_—absorbance of control (cells treated with fresh culture medium).

According to the International Standard ISO 10993-5:2010, a reduction in cell viability of more than 30% is considered a potential cytotoxic effect.

### 2.4. Statistical Analysis

The MTT assay was performed in three independent experiments with six replicates for each concentration of tested material. Data were expressed as means ± standard deviation. IBM SPSS Statistics for Windows (Version 23.0. Armonk, IBM Corp, Armonk, NY, USA) software was used for the statistical analysis. An analysis of variance (ANOVA) and Dunnett’s post hoc tests were performed. All tests were performed at the 0.05 significance level. If the *p*-value was less than 0.05, the differences were considered statistically significant.

## 3. Results

The aim of this in vitro study was to evaluate the level of the cytotoxic effect of new resin-modified calcium silicate and calcium phosphate materials using the mouse fibroblast Balb/3T3 cell line. The MTT assay was used, and cell viability was evaluated. The greater the reduction in cell viability, the greater the cytotoxic effect.

**Figure 7:**
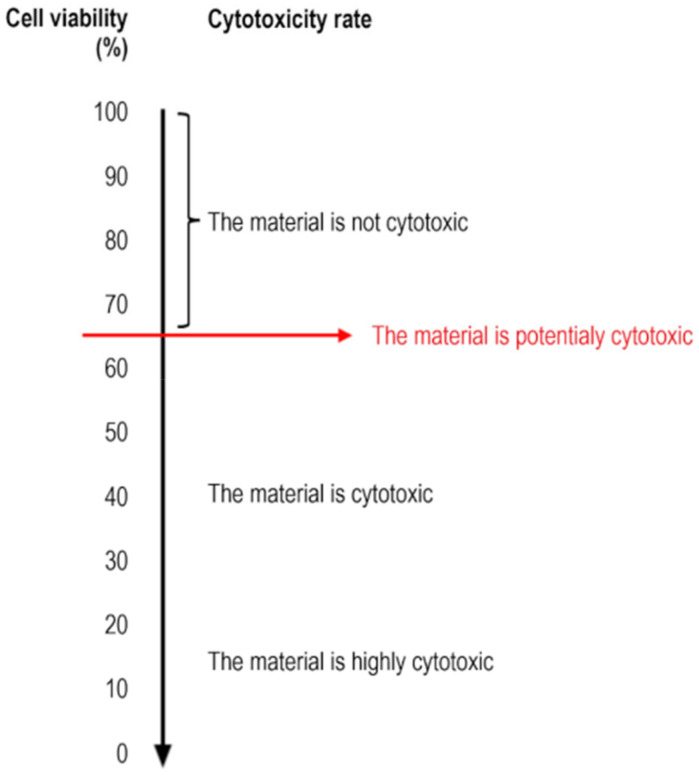
Relationship between the cell viability and the cytotoxicity rate.

According to the International Standard ISO 10993-5:2010, a decrease in cell viability of more than 30% is considered a potential cytotoxic effect (Figure 7).

**Figure 8:**
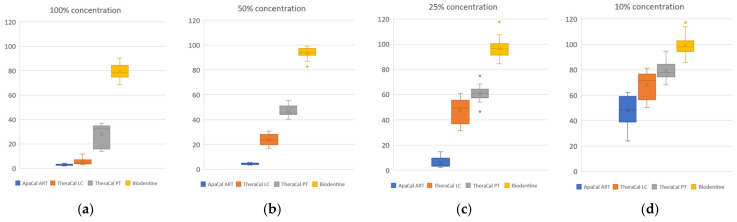
Distribution of quantitative values of cell culture viability for all tested materials: (**a**) 100% extract concentration; (**b**) 50% extract concentration; (**c**) 25% extract concentration; (**d**) 10% extract concentration.

The culture viability (%) was calculated for six replicates of each concentration of tested material, and the distribution of these quantitative values is shown as box graphs in Figure 8a–d.

**Figure 9:**
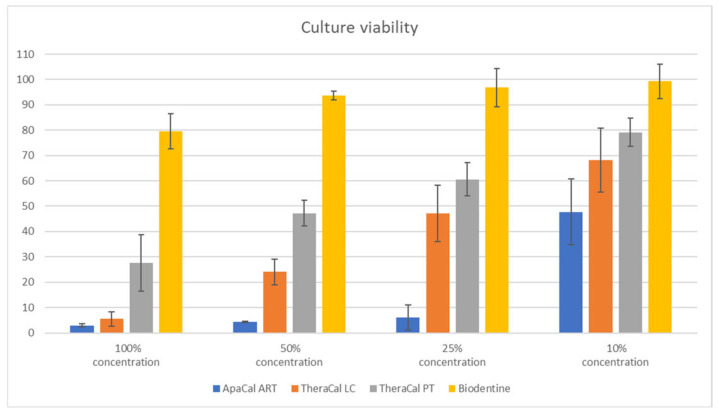
Cell viability (mean (%) and std. deviation) for all concentrations of the tested materials.

For the culture viability at each concentration of all investigated samples, the mean (Table 2) and standard deviation were expressed from all three independent tests (Figure 9)

Comparison of four groups according to material was performed using an analysis of variance (ANOVA). The values for individual materials were statistically significantly different (Table 3).

Subsequently, Dunnett’s post hoc tests were performed, which compared the investigated materials to the reference material Biodentine. It was shown that, at all concentrations, the cell viability was statistically significantly lower for the studied materials compared to Biodentine. Only the difference for TheraCal PT at a concentration of 10% was statistically insignificant (Table 4).

According to these processed data, it is clear that Biodentine can be considered a non-cytotoxic material at all of the investigated concentrations. On the other hand, ApaCal ART shows a high level of cytotoxicity at all tested concentrations (cell viability reduction of more than 50%). TheraCal LC in concentrations of 100%, 50%, and 25% can be considered cytotoxic (decrease in cell viability of more than 50%). However, at a concentration of 10%, the cell viability was reduced by an average of 32%; therefore, it can be considered only potentially cytotoxic at this concentration. TheraCal PT can also be described as cytotoxic at concentrations of 100% and 50%, as the viability of the culture decreased by more than 50%. On the contrary, at a concentration of 25%, the reduction in cell viability did not exceed 40%, indicating only potential cytotoxicity. Moreover, at a concentration of 10%, the cell viability was, on average, 79.18% (the reduction did not exceed 30%); thus, TheraCal PT can be considered non-cytotoxic at this dilution, similar to Biodentine.

## 4. Discussion

As the main disadvantages of first-generation calcium silicate cements [20] include difficult manipulation and a relatively long setting time [17], manufacturers try to invent modifications that eliminate these problems. When using one of the vital pulp therapy procedures, it is necessary to hermetically close the cavity immediately. Thus, it is even more important to use a type of the material that can chemically set within minutes (second-generation [20] or third-generation material with a putty-like consistency [20,38]) or can be light-cured, so that subsequent definitive hermetical reconstruction is made possible. For materials to be light-cured, they must be resin-modified. Since composite materials are not recommended for direct contact with the dental pulp [2,10], it is possible to assume that the biocompatibility of the resin-modified bioceramic materials may also be decreased.

The aim of this in vitro study was to evaluate the cytotoxic effect of three different groups of resin-modified materials: 1. Light-cured resin-modified calcium silicate cement (the selected material was TheraCal LC), 2. Dual cured resin-modified calcium silicate cement (the selected material was TheraCal PT), and 3. Light-cured resin-modified calcium phosphate cement (the only material available was ApaCal ART). These materials were compared with the classic calcium silicate cement Biodentine, which is commonly used in vital pulp treatment.

All of these materials consist mainly of filler (dicalcium silicate, tricalcium silicate, or calcium phosphate), which is expressed in different amounts and ratios in each material. The radiopaque filler (e.g., zirconium oxide, barium zirconate, ytterbium fluoride) is another component that is present in all investigated materials [23,39]. When comparing the composition of individual materials, it is clear that the main difference between classic calcium silicate cements and modified materials is the presence of the resin component (e.g., Urethane Dimethacrylate, Bis-GMA, PEGDMA), stabilisers, and initiators [32]. Unfortunately, the exact amount of these ingredients is not declared in all of these materials (although dissimilar fractions may affect biocompatibility differently). The manufacturer of TheraCal LC and TheraCal PT states that 5–10% of Bis-GMA is included; on the other hand, the manufacturer of ApaCal ART does not provide any specific information about the ratio of the components.

According to the MTT assay, it has been verified that Biodentine has no cytotoxic effect on the cells at any concentration after 24 h (the cell viability was at least 79.49%, and the microscopic image of the concentration of 100% is mostly comparable with the negative control). This result is comparable to those of previously published studies [40,41].

In contrast, the resin-modified calcium phosphate material ApaCal ART showed the greatest cytotoxicity level at all concentrations (also, the microscopic image of the concentration of 100% is more similar to the positive control). The null hypothesis is rejected, as the *p*-values are ≤0.001 at all concentrations. Therefore, these results verify the alternative hypothesis. It cannot be compared with previous studies because the necessary data have not been published yet. Based on the high cytotoxic effect as a result of this in vitro study, it can be assumed that the material is not primarily suitable for direct contact with the dental pulp. However, it is still possible to use it as an option in the case of indirect pulp capping due to the advantages mentioned in the introduction, but more detailed studies comparing other properties of this material still need to be carried out.

On the other hand, literature focusing on the cytotoxic effect of TheraCal LC has already been published in recent years. The methodology was similar in these studies, with the main difference being the use of different cell cultures (the rat odontoblast-like cell line [25], human dental pulp stem cells [26,34]). In our in vitro study, the cell viability of the cultures at all concentrations was significantly reduced in this material, and this result is comparable to those of previous studies. According to the results, TheraCal LC can be described as potentially cytotoxic at the maximum dilution (10%) and highly cytotoxic at higher concentrations of the material (equally, the microscopic image at 100% concentration is similar to the positive control). This result is comparable to those of previous studies [25,26,34]. As the *p*-values are ≤0.013, the null hypothesis is rejected and the alternative hypothesis is verified. Due to this fact, it should not be recommended for direct contact with the dental pulp. However, as it is user-friendly in terms of handling and the cited literature shows that its results are significantly better than common base cements (Dycal, Calcimol LC) [25,26], it is still preferable to only use it in the indication of indirect pulp capping.

As the material TheraCal PT was introduced into the dental market less than five years ago, its biological properties have been described in only a few studies. The cytotoxicity effect was evaluated in two studies [27,34], and the findings are comparable to the results of this in vitro study. The cytotoxicity level in the case of TheraCal PT was high at a concentration of 100% (however, the cell viability was 27.63%; this result was better than for ApaCal ART and TheraCal LC, and the microscopic image at the 100% concentration also corresponds with this result) and 50%. However, with gradual dilution, the cytotoxic effect significantly decreased. At a concentration of 10%, it could be characterised as a material that is not potentially cytotoxic, which is comparable to the reference material Biodentine. The *p*-value of this concentration is 0.095, so this verifies the null hypothesis for the concentration of 10%. However, the *p*-values for higher concentrations are ≤0.001; thus, the null hypothesis is rejected in these cases and the alternative hypothesis is verified. Likewise, these results are similar to those of previous in vitro studies, where the highly diluted dual-cured material showed the same results as the pure calcium silicate material [27,34].

This independent study was carried out primarily due to the inconsistency and non-standardisation [42] of previous studies (each study used different cell lines or methodologies; in addition, the group of light-cured resin-modified calcium phosphate cements has not been researched yet). Therefore, there was a necessity to conduct a study that could compare all of these groups of materials using one standardised methodology.

However, this study has also some limitations.

The first limitation may be the use of the mouse fibroblast cell line, whereas some of the previous studies focused on TheraCal LC and TheraCal PT were performed on the odontoblast-like cell line or human dental pulp stem cells [25,26,34].

Another limitation of this and previous studies [27,34] might be the different interpretation of the results. Extracts of the materials are investigated in several dilutions; however, for clinical practice, the most important results are at a concentration of 100%. For this reason, the conclusion of our study might differ from those of previous publications, where the result was related to the maximum dilution of the extract [16,30].

The last limitation of this study is the fact that the material extract is in contact with the entire cell culture in the methodology used. However, it is necessary to realise that in clinical practice, the contact with the material is only on the exposed surface of the dental pulp. The main questions are as follows: How deep in the dental pulp tissue does the cytotoxic effect occur and cause tissue damage? Can the material induce a defensive reaction in the dental pulp? A study evaluating the biocompatibility and regeneration of the dentin–pulp complex in vivo was published, where TheraCal PT was evaluated in comparison with calcium silicate cements. An inflammatory infiltrate was present in all groups, despite there also being very similar reparative dentin formation in all of the observed groups [43]. However, for the other tested materials in our in vitro study, and for a better understanding of the whole issue, more detailed research is needed. Conducting randomised clinical studies and cohort studies on this topic is recommended.

To summarise the results and their clinical relevance for general dentists, the null hypothesis that the resin-modified cements show the same level of cytotoxicity as classic calcium silicate cements was rejected in all cases, except for the TheraCal PT at a concentration of 10%. Thus, the alternative hypothesis, that all groups of resin-modified materials (calcium silicate or calcium phosphate materials) show higher cytotoxic effects compared to classic calcium silicate cement, was verified in all cases except for TheraCal PT at the 10% dilution. ApaCal ART in any concentration might not be suitable for direct contact with the dental pulp, as its possible cytotoxic effect may cause the loss of the vitality of the tooth. Since TheraCal LC at low concentrations shows similar properties to the reference material and TheraCal PT at the lowest concentration shows the same properties as Biodentine, these materials can be considered as potentially suitable for direct contact with the dental pulp. This result is comparable with anther other study [27]. However, it is necessary to note that, in clinical practice, the materials are only used at a 100% concentration and no dilution is possible. For this reason, neither TheraCal LC nor TheraCal PT appear to be suitable materials primarily indicated for direct contact with the dental pulp according to the results of this in vitro study. Nevertheless, in order to make definitive clinical recommendations for each of these groups of materials, in vivo studies based on the histological examination of the treated dental pulp should be conducted.

## 5. Conclusions

For clinical use, it is more appropriate to continue using classic calcium silicate materials for procedures that preserve the vitality of the dental pulp. Calcium silicate and calcium phosphate materials that are resin-modified can be primarily used for indirect pulp capping or stepwise excavation, but due to their significantly higher cytotoxic potential at a concentration of 100%, it is recommended that they are used with caution when in direct contact with the vital dental pulp.

## Figures and Tables

**Table 1:** The composition of the tested materials.

Material	Form	Manufacturer’s Brochure	Composition According to Literature
ApaCal ART	Premixed syringe	Urethane Dimethacrylate, Triethylene Glycol Dimethacrylate, Calcium Hydroxide, Tricalcium Phosphate, Hydroxyapatite, Photoinitiators, Stabilisers, Radiopacifiers	Urethane Dimethacrylate, Triethylene Glycol Dimethacrylate, Calcium Hydroxide, Tricalcium Phosphate, Hydroxyapatite, Photoinitiators, Stabilisers, Barium Zirconate [32]
TheraCal LC	Premixed syringe	Resin-modified Calcium Silicate	Calcium Silicate (Portland cement type III) (30–50%), Bis-GMA (Bisphenol A Diglycidyl Meth-acrylate) (5–10%), PEGDMA (Polyethylene Glycol Dimethacrylate), Barium Zirconate (1–5%), [32,33] Strontium Glass, Fumed Silica) [34]
TheraCal PT	Dual syringe with automix tip	Resin-modified Calcium Silicate	SG-Mix cement (50–75%), Bis-GMA (5–10%), Barium Zirconate (1–5%), Ytterbium Fluoride (1–5%), Initiator (<1%) [34]
Biodentine	Powder capsule and liquid ampule (1 powder capsule: 1 single-dose container of liquid)	Tricalcium Silicate powder; Aqueous Calcium Chloride solution Excipients	Powder: Tricalcium Silicate (80.1%), Dicalcium Silicate, Calcium Carbonate (14.9%), Zirconium Oxide (5–10%); Liquid: Water, Calcium-Chloride-Modified Polycarboxylate (10–25%) [23,33,35]

**Table 2:** Cell viability for all concentrations of the tested materials (non-cytotoxic effect is marked by green colour).

Material Extract	100% Concentration	50% Concentration	25% Concentration	10% Concentration
ApaCal ART	2.98	4.34	6.12	47.71
TheraCal LC	5.54	24.05	47.22	68.18
TheraCal PT	27.63	47.24	60.63	79.18
Biodentine	79.49	93.67	96.79	99.29

**Table 3:** ANOVA comparison of the mean values of the tested materials (statistically significant difference (*p* < 0.05) is marked by red colour).

Extract Concentration	Material	N	Culture Viability		*p* (ANOVA)
			Mean	Std. Deviation	
100%	ApaCal ART	3	2.98	0.60	<0.0001
	TheraCal LC	3	5.54	2.83	
	TheraCal PT	3	27.63	11.04	
	Biodentine	3	79.49	6.88	
50%	ApaCal ART	3	4.34	0.22	<0.0001
	TheraCal LC	3	24.05	5.15	
	TheraCal PT	3	47.24	5.06	
	Biodentine	3	93.67	1.75	
25%	ApaCal ART	3	6.12	4.96	<0.0001
	TheraCal LC	3	47.22	11.11	
	TheraCal PT	3	60.63	6.67	
	Biodentine	3	96.79	7.58	
10%	ApaCal ART	3	47.71	13.03	0.002
	TheraCal LC	3	68.18	12.51	
	TheraCal PT	3	79.18	5.60	
	Biodentine	3	99.29	6.85	

**Table 4:** Comparison of the values of each tested material with the reference material Biodentine, according to Dunnett’s post hoc tests (statistically significant difference (*p* < 0.05) is marked by red colour).

Extract Concentration	Material	Reference Material	Mean Difference	*p*
100%	ApaCal ART	Biodentine	−76.51667	<0.0001
	TheraCal LC	Biodentine	−73.95333	<0.0001
	TheraCal PT	Biodentine	−51.86000	<0.0001
50%	ApaCal ART	Biodentine	−89.32667	<0.0001
	TheraCal LC	Biodentine	−69.62333	<0.0001
	TheraCal PT	Biodentine	−46.43000	<0.0001
25%	ApaCal ART	Biodentine	−90.66667	<0.0001
	TheraCal LC	Biodentine	−49.56667	0.0002
	TheraCal PT	Biodentine	−36.15333	0.001
10%	ApaCal ART	Biodentine	−51.58000	0.001
	TheraCal LC	Biodentine	−31.11000	0.013
	TheraCal PT	Biodentine	−20.11333	0.095

## Data Availability

The original contributions presented in the study are included in the article, further inquiries can be directed to the corresponding author.
